# Efficacy and safety of tocilizumab (TCZ) in patients with systemic juvenile idiopathic arthritis (SJIA): tender 52-week data

**DOI:** 10.1186/1546-0096-9-S1-P164

**Published:** 2011-09-14

**Authors:** F De Benedetti, H Brunner, N Ruperto, R Schneider, P Woo, R Burgos-Vargas, P Dolezalova, R Joos, N Wulffraat, Z Zuber, F Zulian, R Allen, D Brown, J Chaitow, M Pardeo, G Espada, B Flato, V Gerloni, G Horneff, S Wright, A Kenwright, D Lovell, A Martini

**Affiliations:** 1 Ospedale Bambino Gesù, Rome, Italy; 2 PRCSG, Cincinnati, USA; 3 PRINTO, Genoa, Italy; 4 Roche, Welwyn, UK

## Background

sJIA refractory to immunosuppressants including methotrexate and TNF-α inhibitors can lead to severe disabilities. Excessive IL-6 production has been implicated in the pathoetiology of sJIA.

## Aim

To determine the efficacy and safety of TCZ, an IL-6 receptor inhibitor, in patients (pts) with sJIA treated for 52 weeks (wks) in the ongoing, 3-part, 5-year, phase 3 TENDER study.

## Methods

Pts (N=112) 2–17 years with active sJIA for ≥6 months were randomized 2:1 to TCZ (8 mg/kg if body weight ≥30 kg; 12 mg/kg if <30 kg) or placebo every 2 wks for 12 wks in part 1; all pts received open-label (OL) TCZ in part 2. Pts who escaped to OL TCZ in part 1 also entered part 2. Oral corticosteroid (CS) tapering was permitted at wks 6 and 8 in part 1 and in the OL extension in the presence of ACR70 response, ESR <20 mm/h, and absence of fever. Efficacy data included pts who reached wk 52 of TCZ treatment (n=88); safety data considered all pts (N=112).

## Results

Proportions of TCZ pts who achieved JIA ACR30 + absence of fever or JIA ACR70/90 increased to wk 52 (Table). Number of joints with active arthritis or with limitation of movement decreased from 19.8±15.7 and 19.8±15.6, respectively, to 3.0±7.0 and 7.5±11.7 at wk 52 (45% of pts had 0 active joints). CHAQ-DI score improved from 1.7±0.9 to 0.7±0.8 at wk 52. Physician global assessment VAS and pt/parent global assessment VAS improved from 64.9±22.3 and 58.7±24.4, respectively, to 9.7±12.8 and 12.6±18.5 at wk 52. CS dose decreased from 0.30±0.20 mg/kg/d to 0.06±0.08 at wk 52; 48% discontinued CSs. 33 serious AEs (SAEs) occurred in 25 pts; 12 SAEs were considered related (remotely, possibly, or probably) to TCZ (rate: 0.23/pt year [PY] in part 1, 0.25/PY in part 2). 15 serious infections occurred; 6 (gastroenteritis, varicella, septic arthritis, otitis media, pharyngotonsillitis, upper respiratory tract infection) were considered related to TCZ; all resolved and none led to discontinuation. 12 pts withdrew (4, AEs; 4, insufficient response). One pt died of a suspected tension pneumothorax considered unrelated to treatment.

## Conclusions

TENDER 1-year results demonstrate that TCZ is highly effective and generally well tolerated in pts with sJIA.

**Table:** 

	Wk 12		Wk 52
**Responses**	**Placebo (n=37)**	**TCZ (n=75)**	**TCZ (n=88)**

JIA ACR30 + absence of fever, n (%)	9 (24)	64 (85)	77 (88)
JIA ACR70, n (%)	3 (8)	53 (71)	78 (89)
JIA ACR90, n (%)	2 (5)	28 (37)	57 (65)

